# Amniotic Fluid Stem Cells and Their Application in Cell-Based Tissue Regeneration

**Published:** 2012-12-17

**Authors:** Mohamadreza Baghaban Eslaminejad, Shahrbanoo Jahangir

**Affiliations:** Department of Stem Cells and Developmental Biology, Cell Science Research Center, Royan Institute for Stem Cell Biology and Technology, ACECR, Tehran, Iran

**Keywords:** Amniotic Fluid, Stem Cells, Mesenchymal Stem Cells, Tissue Regeneration, Tissue Engineering

## Abstract

Advances in stem cell biotechnology hold great promise in the field of tissue engineering and regenerative medicine. Of interest are marrow mesenchymal stem cells (MSCs), embryonic stem cells (ESCs), and induced pluripotent stem cells (iPSCs). In addition, amniotic fluid stem cells (AFSCs) have attracted attention as a viable choice following the search for an alternative stem cell source. Investigators are interested in these cells because they come from the amniotic fluid that is routinely discarded after birth. There have been multiple investigations conducted worldwide in an attempt to better understand AF-SCs in terms of their potential use in regenerative medicine. In this review we give a brief introduction of amniotic fluid followed by a description of the cells present within this fluid. Their history related to stem cell discovery in the amniotic fluid as well as the main characteristics of AF-SCs are discussed. Finally, we elaborate on the potential for these cells to promote regeneration of various tissue defects, including fetal tissue, the nervous system, heart, lungs, kidneys, bones, and cartilage.

## Introduction

Regenerative medicine is an emerging, rapidly advancing field in therapeutics and research. This new medical discipline focuses on replacing or regenerating human cells, tissues, and organs that are largely damaged. The repair of damaged cells in the body is accomplished by stem cells residing in tissues. Regenerative medicine tries to harness the power of stem cells as well as the body’s own regenerative capabilities to restore function to lost or defective tissue. In addition to the study of stem cells, regenerative medicine includes the field of tissue engineering (1 , 2 ).

Tissue engineering is an interdisciplinary science with considerable potential for promoting regenerative medicine. Tissue engineering consists of three building blocks that include a scaffold (ECM material) that can be constructed from polymers, ceramics and composites, cells and growth factors. Various scientific fields (physics, chemistry, engineering, material sciences, biology, and medicine) are involved in the provision of scaffolds, cell preparation, determination of growth factor types, and most importantly, combining these components to create the desired constructs that comprise a functional tissue (3 , 4 ). One challenge in tissue engineering is to locate a reliable cell source. Stem cells are considered promising candidates for tissue engineering and regenerative medicine because of their extensive self-renewal property and multi-lineage differentiation capacity. To date, several stem cell types have been introduced with potential application in regenerative medicine. Of these, mesenchymal stem cells (MSCs), embryonic stem cells (ESCs), and induced pluripotent stem cells (iPSCs) have gained extensive attention.

MSCs are a subtype of adult stem cells (ASCs) defined as undifferentiated cells found throughout the body after birth (5 ). Other types of ASCs include hematopoietic stem cells (6 ), neural stem cells (7 ), endothelial progenitor cells (8 ), intestinal stem cells (9 ), olfactory stem cells (10 ), and testicular stem cells (11 , 12 ). These cells are able to divide and replace dying cells and regenerate injured tissues (5 ).

MSCs are non-hematopoietic cells originally isolated by Friedenstein et al. (13 ). They are an adherent morphologically fibroblastic cell population that resides in marrow tissue. Previous research has shown that the most important characteristic of MSCs is their capacity to produce small deposits of bone and cartilage-like tissue in culture (13 , 14 ). MSCs are also able to give rise to neurons, keratinocytes, lung epithelial cells, liver cells, intestine epithelial cells, and kidney and spleen cells, in addition to their well-recognized capacity to differentiate into skeletal cell lineages (15 -17 ).

This property of MSCs in producing cells other than mesenchymal cell lineages is referred to as MSCs transdifferentiation (5 , 18 ). The efficacy of MSCs in treating some tissue defects is well established. Namely, MSCs have been used to cure osteogenesis imperfecta, regenerate bone tissue defects, reconstruct cardiac muscle after infarction, resurface articular cartilage, and to restore hematopoiesis in patients undergoing chemotherapy (19 -23 ). Despite these outstanding achievements with MSCs, the following disadvantages exist: i. the invasiveness of obtaining cells from marrow which involves insertion of a needle into the patient’s iliac crest in order to aspirate the marrow sample, ii. the number of MSCs is limited in marrow tissues and is even less in elderly people, and iii. MSCs undergo senescence in culture (24 , 25 ).

ESCs are pluripotent cells derived from a blastocyst inner cell mass (26 ). They possess indefinite self-renewal potential and have the capability of differentiation to all three germ layers. Despite these prominent properties, ESCs are still not applicable as cellular material in regenerative medicine because of various concerns, including immunologic incompatibility, the possibility of teratoma formation in transplantations, and certain ethical issues (27 ). Alternatively scientists have attempted to establish ESC-like stem cells, known as iPSCs, from somatic cells (28 ). Although iPSCs have some advantages over ESCs, the production of iPSCs through plasmid or adenovirus-based transduction is a main concern towards their application in the cell-based treatment of tissue defects (28 ).

Thus, the attention of investigators has been directed to easily attainable sources, such as peripheral and umbilicalcord blood (29 -31 ). Another source for stem cells would be amniotic fluid, which possesses several advantages as cellular material for cell-based treatment of tissue defects. Amniotic fluid can easily be collected through a safe procedure (amniocentesis) that is routinely performed for the prenatal diagnosis of genetic diseases, its stem cells are not tumorigenic after transplantation, and obtaining amniotic fluid during pregnancy is neither harmful to the mother nor to the fetus (32 ).

### Amniotic fluid

Amniotic fluid is a protective, nourishing fluid that surrounds the embryo during pregnancy. This fluid starts to gather immediately after formation of the amniotic cavity (33 ). The average volume is 270 ml at week 16 which increases to 400 ml at week 20 of pregnancy. During the first half of the pregnancy amniotic fluid is secreted mainly as a result of active transport of sodium and chloride, which is accompanied by transport of water through the chorio-amniotic membrane and embryo's skin (34 ). During the second half of pregnancy, the production of urine and respiratory fluid both contribute to the volume of amniotic fluid (35 , 36 ).

At this time the embryo begins to intake amniotic fluid and returns it into amniotic sac through the digestive as well as the urinary tracts. The composition and dynamics of amniotic fluid varies according to the stage of pregnancy (37 , 38 ). In general the fluid contains proteins, carbohydrates, fats, amino acids, enzymes, hormones, pigments, and cells. In humans during the early days of pregnancy amniotic fluid is isotonic but after the keratinization of the embryo's skin, which usually occurs at week 24 of pregnancy, the fluid become hypotonic (39 , 40 ).

### Cells present in amniotic fluid

Amniotic fluid is in contact with various embryonic components. On one side it is in contact with the embryo's skin and amniotic membrane, whereas on the other side it is exposed to the embryonic digestive tract and the embryonic urinary and respiratory ducts. Investigations have demonstrated that a variety of embryonic cells, including those from the three embryonic germinal layers, are present in amniotic fluid (41 -43 ). These cells are probably released from the amniotic membrane, embryonic skin, digestive tract, and the respiratory and urogenital systems. In addition to fully differentiated cells the presence of precursor and multi-potent stem-like cells have also been described within amniotic fluid (44 , 45 ). According to reports, the characteristics of amniotic fluid cells are varied based on gestational age and embryo pathology (46 ).

*In vitro* studies have indicated that the majority of amniotic fluid cells are non-adherent. Adherent cells are estimated to comprise 5-8×10^5^ cells/l of amniotic fluid. Approximately 5% of the adherent cells are morphologically small, rounded cells (47 , 48 ). The total nucleated cells in each ml of amniotic fluid varies between 10^3^-10^6^. The cells capable of forming a colony are rarely present. According to research, cells isolated from amniotic fluid at weeks 16-18 of pregnancy could only produce an average of 3.5 ± 1.8 colonies/ml of amniotic fluid at day 12 of culture (41 ).

Based on morphologic and growth characteristics, amniotic fluid cells can be divided into the following cell groups: epithelial (E-type), amniotic fluid (AF-type), and fibroblast (F-type). Both E-and AF-type cells appear during the early days of amniotic fluid cell culture, AF-type cells remain throughout the culture period while E-type cells soon disappear. F-type cells usually appear during late primary culture and possess phenotypic and differentiation characteristics similar to marrow MSCs (41 -43 ).

### Progenitor and stem cells in amniotic fluid

According to Tsai et al. a variety of human cells in amniotic fluid are shed from embryonic and extraembryonic tissues during the process of fetal development and growth (49 ). Progenitor cells in amniotic fluid were initially discovered in 1993 when small nucleated cells of a round morphology, similar to the hematopoietic precursor, were recognized in amniotic fluid obtained from a woman 12 weeks pregnant. These cells probably originated from the yolk sac (50 ). In 1996 it was reported that the amniotic fluid also contained a population of nonhematopoietic progenitors that had the capability to differentiate into myogenic cell lineages (51 ).

Prusa et al. isolated pluripotent stem cells from amniotic fluid collected during week 14 of pregnancy in an attempt to analyze human amniotic fluid samples for expression of Oct-4, stem cell factor (SCF), vimentin, and alkaline phosphatase. They found a population of Oct-4-expressing cells in the fluid (44 ). In the same year In’t Anker et al. also reported that amniotic fluid from week 19 of pregnancy contained a population of MSCs (52 ). In 2004, Tsai et al. succeeded in isolating MSCs from amniotic fluid from weeks 16-20 of pregnancy. Isolated cells have been reported to possess a high proliferation rate and the capability for differentiation into adipocytes, osteocytes, and neurons (49 ). The neurogenic differentiation of amniotic stem cells from fluid at weeks15-17- of pregnancy was later confirmed by other studies (53 , 54 ). In 2007, De Coppi et al. reported the isolation of rodent amniotic fluid-derived stem cells from a C57BL/6J mouse at day 11.5 of pregnancy with highly interesting characteristics. According to their reports, the cells tended to express embryonic and adult stem cell markers. Unlike ESCs the cells were able to expand in culture conditions without the need of a feeder layer and had no tumorigenic activity. One important feature of the cells was their normal karyotype and long telomere, even after extensive propagation (over 250 population doublings) (55 ).

### Isolation of stem cells from amniotic fluid

To obtain stem cells from amniotic fluid, the fluid must first be collected and transferred to a culture lab. Usually amniotic fluid is collected from murines at week 2 of pregnancy (55 ) and from humans during the second (49 ) or third trimester, and sometimes immediately after birth (56 ). At least 2 ml of the fluid must be collected (49 ). The cells are isolated by taking advantage of their ability to adhere to culture surfaces. For stem cell isolation, fluid is mixed with an equal volume of culture medium, usually Dulbecco’s Modified Eagle Medium (DMEM) supplemented with fetal bovine serum (FBS) and antibiotics. This mixture must be placed in culture flasks in an atmosphere of 5% CO_2_ and 37°C. A morphologically heterogeneous cell population appears several days after culture initiation. Fibroblastic cells usually dominate the culture after several round of sub-culturing (57 , 58 ).

### Characteristics of amniotic fluid stem cells (AF-SCs)

One interesting feature of amniotic stem cells is the presence of telomerase activity. Telomerase is an enzyme that maintains telomere sequences at chromosomal ends. This sequence protects the end of the chromosome from deterioration or from fusion with neighboring chromosomes. Telomerase activity is a known marker of human pluripotent stem cells, embryonic proliferating cells, and germ cells (46 ). Since human somatic cells contain no telomerase activity their telomere length becomes progressively shorter after each division. In 1999 it was found that amniotic fluid cells have telomerase activity, which was observed in both cultured and uncultured cells (59 ). Studies conducted by our group were also in accordance with this observation (57 , 58 ). Another interesting finding was the discovery of Oct-4 expression on amniotic stem cells. According to previous investigations embryonic carcinoma cells, ESCs, and embryonic germ cells tended to express the Oct-4 marker (60 , 61 ). Research studies have shown the presence of a population of Oct-4-positive cells in amniotic fluid (62 ).

Amniotic fluid cells share some features with MSCs and ESCs regarding the expression of some marker genes. According to research, amniotic fluid stem cells (AF-SCs) express MSC markers CD90, CD105, CD73, CD44, and CD29. Similar to MSCs, they do not express hematopoietic markers such as CD45, CD34, and CD133. With regards to the expression of immunogenic markers, amniotic stem cells behave like MSCs and express MHC II at a very low level (52 , 63 , 64 ).

Amniotic stem cells are similar to ESCs in terms of some markers. SSEA-4, which is expressed in ESCs but not MSCs, is also expressed in AF-SCs (65 ). It has been found that the genes of SCF (a pluripotent marker), CK18, and nestin are expressed in fibroblastic cells from amniotic fluid (46 ). Amniotic stem cells also express vimentin and alkaline phosphatase, which are markers of pluripotent stem cells (44 ). It should be mentioned that despite these similarities differences exist between amniotic stem cells and ESCs. In contrast to ESCs , AF-SCs do not express SSEA-3 and Tra-1-81. Most AF-SCs weakly express Tra-1-60. Table 1 summarizes the presence and absence of some markers for MSCs, ESCs, AF-SCs, and AF-MSCs.

In the literature, amniotic-derived cells that contain stem cell characteristics have been referred to by two nominations: AF-SCs and AF-MSCs.

**Table 1:** Expression of some markers at varying stem cells


Surface markers	MSCs	ESCs	AF-SCs	AF-MSCs	Ref

**CD90**	+		+	+	(47, 49, 66-67)
**CD105**	+			+	(49, 66-68)
**CD73**	+			+	(66, 68)
**CD44**	+		+	+	(47, 49, 66-68)
**CD29**	+		+	+	(66-68)
**CD45**	-		-	-	(66-68)
**CD34**	±		-	-	(47, 49, 66-68)
**CD31**	-		-	-	(68-69)
**MHSCII**	-		-	-	(47, 49, 66, 68)
**SSEA-4**		+	+		(69-70)
**SCF**	+	+		+	(46)
**CK18**	+			+	(46)
**Nestin**	+		+	+	(46-47)
**Alkaline Phosphatase**		+			(70)
**SSEA-3**		+			(70)
**Tra-1-81**		+	+		(69-71)
**Tra-1-60**		+	+		(69, 71)
**Oct-4**		+	+		(69, 70)


+; Positive, -; Negative, ± ; Weakly positive,and Blank; No report.

### Amniotic fluid stem cells in tissue engineering and regeneration

Several researchers have attempted to fabricate engineered constructs using AF-SCs with the intent to promote regeneration in tissue defects created in animal models. We will briefly review these studies.

### Fetal tissue reconstruction

One interesting application of amniotic stem cells is in the field of fetal tissue engineering, as suggested by Kaviani et al. (72 ). These authors have seeded a subpopulation of mesenchymal cells from the amniotic fluid onto polyglycolic acid/ poly-4-hydroxyapatite scaffolds and observed that the cells were able to attach firmly to the scaffolds and form confluent layers with no evidence of cell death.

Several years later, following this suggestion, Kunisaki et al. (73 ) engineered a construct using mesenchymal amniocytes and scaffolds which was subsequently transplanted into an experimentallycreated diaphragmatic defect in neonatal lambs. The scaffold was composed of trilayered composites made of 70% type I collagen hydrogel solution placed between a cellular human dermis and single-ply small intestinal submucosa. To hold the three layers together, peripheral simple interrupted sutures of 5-0 monofilament polypropylene were used. According to a report by Kunisaki et al., the diaphragmatic defect was repaired with a mesenchymal amniocyte-based construct leading to improved structural outcomes when compared with equivalent fetal myoblast-based grafts. These authors suggested that the amniotic fluid could be a good cell source for tissue engineered diaphragmatic reconstruction (74 ).

### Regeneration of neural tissue

Amniotic MSCs have been reported to be able to promote regeneration in central nervous tissue. Cipriani et al. explored the fate of AF-MSCs after transplanting the cells into the striatum of normal and ischemic rats. They noticed that the grafted cells tended to survive and migrate towards injured brain regions in the ischemic animals and towards several regions in normal animals. Immunohistochemical analysis showed that the cells had differentiated into neurons as well as astrocytes. They suggested the amniotic fluid could be an alternative source for MSCs (75 ).

The effectiveness of AF-MSCs has also been reported in regeneration of the peripheral nerve (sciatic). In this context the investigation by Pan et al. was remarkable. They prepared a construct by embedding rat AF-MSCs in fibrin glue, which was then delivered in to the crushed sciatic nerve in rat model (76 ). To promote peripheral nerve regeneration, Cheng et al. used a different strategy. They transduced human AF-MSCs with glia cell line-derived neurotrophic factor (GDNF) and embedded them in matrigel. The construct was then transplanted in to the injured sciatic nerve of rats. The results indicated that GDNF-modified human AF-MSCs promoted nerve regeneration. More importantly, GDNF expressed consecutively in the induced cells for up to four weeks (77 ).

Some authors have used combined therapy, i.e. AF-SCs along with some cytokines to promote regeneration of the sciatic nerve. In a study by Pan et al., AF-MSCs were embedded in fibrin glue and the resultant construct was delivered to the injured sciatic nerve. These authors also administrated granulocyte colony stimulating factor (G-CSF; 50 μg/kg) by intraperitoneal injection. According to their results the combination of G-CSF administration and AF-MSCs transplantation led to better outcomes. These authors also have reported that the administration of either AF-SCs, fermented soybean extracts (natto), or combined therapy augments nerve regeneration (78 , 79 ).

### Cardiac regeneration

Others have investigated the application of amniotic stem cells in cardiomyoplasty. Yeh et al. first evaluated human amniotic fluid-derived stem cells to determine if these cells had the capability to give rise to cardiac as well as endothelial cells *in vitro*. Next they created an experimental infarction in a rat model and injected the cells directly into the peri-infarct areas. The results indicated that the injected cells survived and proliferated at the injured site and promoted attenuation of left ventricular remodeling, a higher vascular density, and thus an improvement in cardiac function (80 ).

In another study by the same authors, a cell sheet fragment was fabricated using human AF-SCs and thermo - responsive methylcellulose extracellular matrices. The constructs were transplanted into the peri-ischemic area of an immune-suppressive rat model one week following induction of a myocardial infarction. Transplantation of the cell sheet fragment significantly increased vascular density, improved wall thickness, and also significantly reduced the infarct size when compared with disassociated AF-SCs (81 ).

### Lung epithelial regeneration

AF-SCs have also been used to regenerate lung epithelium. Carraro et al. transplanted human AFSCs into an injured murine lung and investigated their integration and differentiation into pulmonary lineages. They observed that the cells were able to integrate among lung mucosal cells and express alveolar and bronchiolar markers (82 ).

### Kidney regeneration

Research by Perin et al. indicated that AF-SCs were able to be induced to a renal fate in an ex vivo system. These researchers found that injection of AF-SCs into damaged kidney provides a protective effect ameliorating acute tubular necrosis (ATN) in the acute phase in mouse model. They concluded that the cells could represent a novel source of stem cells that may function to modulate the kidney immune milieu in renal failure caused by ATN (83 ).

### Bone and cartilage engineering

AF-SCs have been used in several studies in an attempt to develop a bone construct using tissue engineering principles. Peister et al. investigated the potential of AF-SCs to synthesize the mineralized matrix within porous medical grade poly-epsiloncaprolactone (mPCL) scaffolds (84 ). The cells were cultured within the scaffold and analyzed for their ability to differentiate to osteoblastic cells in the scaffold environment. They observed the deposition of mineralized matrix throughout the scaffold after 15 weeks of three-dimensional (3D) culture. They concluded that AF-SCs were suitable cells to produce 3D mineralized bioengineered constructs both *in vitro* and in vivo, and suggested that the cells were an effective source for functional repair of large bone defects.

Peister et al. cultured AF-SCs on poly-ε-caprolactone and compared them with MSCs cultivated on the same scaffolds. In this study, MSCs differentiated more quickly than AF-SCs, but mineralized matrix produced considerably after five weeks. In contrast, the rate of AF-SC mineralization continued to increase until 15 weeks. They concluded that the stem cell source could dramatically influence the magnitude and rate of osteogenic differentiation *in vitro* (85 ).

The effect of nano scaffolds were also investigated on bone cell differentiation of AF-SCs. Sun et al. cultivated cells onto nanofibrous scaffolds with a morphology similar to that of natural collagen fiber and found that under these conditions there was enhanced alkaline phosphatase activity, calcium content, and the expression of osteogenic genes when compared with traditional scaffolds (86 ).

In a search of better scaffold for bone engineering some authors compared several scaffolds as a matrix of AF-SCs culture. Maraldi et al. have investigated the potential of AF-SCs to synthesize the mineralized matrix within porous scaffolds of collagen, poly-D, L-lactic acid (PDLLA), and silk fibroin in the presence of osteogenic medium. They found fibroin to be an effective scaffold material for the fabrication of a bone construct to functionally repair critical-sized bone defects (87 ).

Some studies developed constructs using scaffolds and transplanted AF-SCs in to animal models to promote regeneration of bone tissue defects. Riccio et al. used human AF-SCs in combination with fibroin scaffolds to repair critical size cranial bone defects in immune compromised rats. Based on confocal analysis that used an antibody directed to a human mitochondrial protein, these authors confirmed the contribution of the transplanted amniotic stem cells in the newly-formed bone at the defect site (88 ).

De Rosa et al. attempted to enhance the osteogenic differentiation of AF-SCs in co-culture setups. They co-cultured AF-SCs with osteocytes derived from dental pulp stem cells (DPSCs) and found that osteoblasts derived from these cells released large amounts of BMP-2 and VEGF into the culture medium. Those morphogenes significantly up-regulated the RUNX-2 gene in AF-SCs (69 ).

According to some reports, AF-SCs can differentiate along chondrocytic cells within a 3D matrix. Kolambkar et al. have investigated the chondrogenic potential of amniotic fluid cells in a pellet as well as a hydrogel culture system and confirmed that they are an alternative source for marrow MSCs for cartilage repair applications (89 ).

## Conclusion

MSCs, ESCs, and iPSCs are among those stem cells with great promise in the field of tissue engineering and regenerative medicine. Because of the disadvantages in using these cells with respect to their applicability in human tissue defects, the search for finding an alternative source of stem cells has led to the discovery of multiple stem cell types. Amniotic fluid is among those sources that recently has gained considerable attention because of their ease of collection, safety in harvesting via amniocentesis both for the mother and fetus, and their inability to form tumors after implantation in vivo. Multiple investigations have thus far been conducted to discover the characteristics of amniotic fluid-derived stem cells and to evaluate the cell potential in regeneration of tissue defects in animal models. The combined data from these studies is promising. However, further investigations are necessary to gain a better understanding of these cells prior to their use in human trials.
